# Constructing the HBV-human protein interaction network to understand the relationship between HBV and hepatocellular carcinoma

**DOI:** 10.1186/1756-9966-29-146

**Published:** 2010-11-16

**Authors:** Zhong-Jun Wu, Yu Zhu, De-Rong Huang, Zhi-Qiang Wang

**Affiliations:** 1 Department of Hepatobiliary Surgery, First Affiliated Hospital, Chongqing Medical University, Chongqing 400016, PR China; 2 Department of Chemistry, Tongji University, Shanghai 200092, PR China

## Abstract

**Background:**

Epidemiological studies have clearly validated the association between hepatitis B virus (HBV) infection and hepatocellular carcinoma (HCC). Patients with chronic HBV infection are at increased risk of HCC, in particular those with active liver disease and cirrhosis.

**Methods:**

We catalogued all published interactions between HBV and human proteins, identifying 250 descriptions of HBV and human protein interactions and 146 unique human proteins that interact with HBV proteins by text mining.

**Results:**

Integration of this data set into a reconstructed human interactome showed that cellular proteins interacting with HBV are made up of core proteins that are interconnected with many pathways. A global analysis based on functional annotation highlighted the enrichment of cellular pathways targeted by HBV.

**Conclusions:**

By connecting the cellular proteins targeted by HBV, we have constructed a central network of proteins associated with hepatocellular carcinoma, which might be to regard as the basis of a detailed map for tracking new cellular interactions, and guiding future investigations.

## Background

Hepatitis B virus is one of the most common infectious diseases in the world, and 43 years after its discovery, it still has a great impact on health, particularly in developing countries. More than 350 million people worldwide are known to be chronic carriers of HBV, and each year 15 million people die of hepatitis [1].

The HBV viral genome is a relaxed-circular, partially duplex DNA of 3,200 base pairs. It has five genes encoding polymerase, pre-S1/pre-S2/S, X protein, precore/core protein, and the ID2828293 gene which is not well understood without an official gene symbol or description[2]. These proteins can also trans-activate other cellular genes, which may play a role in hepatocarcinogenesis [3].

Hepatocellular carcinoma is one of the most common fatal cancers worldwide [4]. HBV is strongly associated with HCC by its presence in the tumor cell and by the striking role of persistent HBV infection as a risk factor for the development of HCC[2]. The incidence of HCC in many countries is increasing in parallel to an increase in chronic HBV infection[1]. It is generally shown that vaccination significantly decreases the incidence of HCC. Moreover, preventing the most severe HBV disease consequences in infected people, such as cirrhosis and fibrosis, will require appropriate therapeutic agents and reduces the risk of developing HCC [5].

To make progress in understanding the mechanisms of viral pathogenesis and the relationship of HCC with HBV, it is important to sort out the interactions of HBV proteins with the vast array of human cellular proteins. While these interactions can be direct viral and host cell protein-protein interactions, many are indirect, including regulatory interactions that alter human gene expression[2]. Rapidly growing knowledge about the protein-protein interaction (PPI) networks (interactome) for hosts and pathogens is beginning to be used to create network-based models [6]. A network analysis approach to a virus-human protein interactome network revealed that host interactors tend to be enriched in proteins that are highly connected in the cellular network [7]. These "hub proteins" are thought to be essential for normal cell functioning and during pathogenesis. Therefore, clarification of the genetic picture of hepatocarcinogenesis caused by HBV infection might provide clues toward achieving a decrease in the incidence of HCC and establishing effective treatments[8].

In this study, we attempted to catalogue all published interactions between HBV and human proteins, particularly human proteins associated with hepatocellular carcinomas, for an in-depth review and understanding of these interactions. Our aim was to enhance insight into HBV replication and pathogenesis on a cellular level, in order to assist in accelerating the development of effective therapeutics.

## Methods

### Text mining of human proteins that interact with HBV and are associated with HCC

To facilitate the development of a database describing HBV and human protein interactions, a detailed literature search was carried out on the PubMed database to analyze binary interactions between HBV and human proteins. We used the automatic text mining pipeline method of NLP (Natural Language Processing), followed by an expert curation process, independent of the results obtained at this step. The data compilation process included publications until January 2009.

In brief, we first searched the document using relevant keywords and transformed it into XML format. We then used the Lingpipe Kit sentence tokenization tool (sentence partition) to separate the abstract text into a single sentence. Follow-up analysis used the sentence as a basic unit. The human genes mentioned in the sentences were extracted using ABNER software [9], and the gene name was normalized based on the Entrez database in order to facilitate analysis and comparison. For example, an extracted conjunction gene description such as "STAT3/5 gene" would be resolved into STAT3 gene and STAT5 gene. We built a protein-protein interaction verb dictionary [10], including terms such as repress, regulate, inhibit, interact, phosphorylate, down-regulate and up-regulate. All of the verbs and their variants were derived from the BioNLP project http://bionlp.sourceforge.net/. Using the Lingpipe Toolkit, we then detected protein interaction verbs in sentences and gathered the HBV protein and synonym names (compiled from the Entrez database). Statistical analysis of sentences in which a human protein, an interaction verb and an HBV protein were all present at the same time (co-occurrence) was used to determine HBV protein and H_HBV _interaction relationships.

Using the same NLP methods, we extracted literature related to hepatocellular carcinoma from PubMed and identified the interactions and relationships between HBV proteins and H_HCC_.

### The integrated human interactome network (H-H network)

In order to make the HBV protein and human protein H_HBV _interaction network more complete, we integrated the H_HBV _and H_HBV _interaction relationships. The H_HBV _and H_HBV _protein interaction data were gathered from the STRING database http://string.embl.de/, which includes experimental evidence of protein interactions (e.g., yeast two-hybrid), protein interaction databases (e.g., the KEGG pathway) and text mining co-occurrence. The algorithm for human protein to human protein interaction relationships was previously described [11]. NCBI official gene names were used to combine protein ACC, protein ID, gene name, symbol or alias from different genome reference databases (e.g., ENSEMBL, UNIPROT, NCBI, INTACT, HPRD, etc.) and to eliminate interaction redundancy due to the existence of different protein isoforms for a single gene. Thus, the gene name was used in the text to identify the protein. Finally, we only used non-redundant protein-protein interactions to build the human interactome data set.

The network structure of the HBV protein to human protein interaction relationships and the human protein to human protein interaction relationships was mapped using Medusa software.

### Gene ontology analysis

To demonstrate the complexity of the HBV-human protein interaction network, the catalogued data were analyzed using gene ontology [12]. Gene ontology is a set of three structured controlled ontologies that describe gene products in terms of their associated cellular component (CC), biological process (BP), or molecular function (MF) in a species-independent manner. We performed gene ontology analysis using EASE software. Enrichment p-values were adjusted by the Benjamini and Hochberg multiple test correction [13].

### Functional analysis using KEGG annotations

Cellular pathway data were retrieved from KEGG, the Kyoto Encyclopedia of Genes and Genomes http://www.genome.jp/kegg/, and were used to annotate NCBI gene functions [14]. For each viral-host protein interaction, the enrichment of a specific KEGG pathway was tested using a Fisher's exact test followed by the Benjamini and Hochberg multiple test correction to control for the false discovery rate [15].

### Network visualization

HBV protein to human protein interaction relationships and human protein to human protein interaction relationships were mapped and visualized in a network structure using Medusa software [16].

## Results

### Construction of an HBV-human interactome network

**Figure 1:**
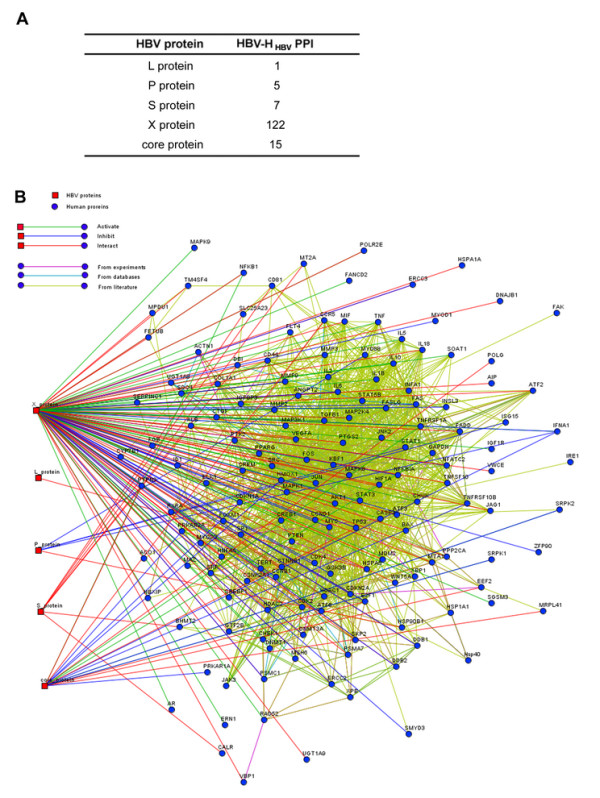
**HBV and human protein interaction network**. (A) Summary of the HBV-human protein (H_HBV_) interactions. (B) HBV and H_HBV _interaction network. Red square: HBV protein. Circular node: H_HBV_. For HBV-H_HBV _interactions, green lines correspond to activate; blue lines, to inhibit; and red lines, to interact (activate or inhibit unknown), all interaction keywords can be found in Additional file 1, Table S2. For H_HBV_-H_HBV _interactions, purple indicates evidence from experiments (High-throughput yeast two-hybrid experiment data was collected from public data sources); light blue, from database (Protein - protein interaction relationship was extracted from KEGG pathway database); and grass green, from literature text mining (Scattered literatures about low throughput research on protein - protein interaction were parsed with an in-house computer program), which derived from the Additional file 1, Table S4.

In order to analyze the interactions between HBV and human proteins, literature indexed in PubMed was searched using keywords [e.g., ("HBV" [title] OR "hepatitis B" [title] AND ("1980/01/01" [PDAT]: "2009/01/01" [PDAT]))]. From the entire database, 52,531 published journal abstracts were identified by NLP (Natural Language Processing) queries. Further text analysis revealed a total of 146 HBV-targeted human protein (H_HBV_) from 250 summary descriptions that reported putative interactions between HBV and human proteins, comprising 150 unique HBV to human protein interactions. Figure 1A summarizes the HBV protein interactions catalogued from these papers (see Additional file 1, Table S1 for a listing of all interactions).

Based on the text in the original journal articles selected by keywords and combining similar keywords, we identified the most important functional keyword used by the authors to describe the interaction. Twenty-five unique keywords were associated with these descriptions. The most frequently used keywords in the database were "interact," 25.77%; "activate," 13.08%; "inhibit," 8.46%; "associate," 9.23%; "regulate," 8.46%, including "upregulate," 3.36%, and "downregulate," 1.54%; and "phosphorylate," 7.31% (Figure 1B, and see Additional file 1, Table S2 for a listing of all keywords). While it could not be excluded that some of these interactions are nonspecific or human errors, the catalogued interactions provide a unique collection of data collectively generated from the available scientific literature.

Analysis of the HBV-infection network showed that X protein and core protein were the most connected proteins (Figure 1A), with 122 (83.5%) and 15 (10.3%) of the total H_HBV _identified in the database, including many transcription factors and regulators. This highlights the potential multi-functionality of these proteins during infection (Figure 1B, Additional file 1, Table S1). Highly interacting proteins are known to be significantly more disordered than low-degree (LD) proteins [17]. Interestingly, X protein and core protein are predicted to contain one intrinsic disordered region (data not shown) according to DISOPRED2 [18]. In addition, five H_HBV _such as MAPK8, E2F1, and NF-kappa 1were targeted by more than one HBV protein, suggesting these proteins might play important roles in HBV replication and pathogenesis (Additional file 1, Table S3).

A human PPI network has been reconstructed from eight databases [19]. This network is composed of 44,223 non-redundant PPIs among 9,520 different proteins, corresponding to 30% of the human proteome (the remaining proteins have no known cellular partners and, therefore, cannot be included in this network). Interestingly, H_HBV _are clearly over-represented in this H-H network (134 (92%) of the total H_HBV_). Analysis of the H_HBV_-H_HBV _sub-network (all connected 146 H_HBV _proteins), which is composed of 1,977 non-redundant PPIs among different H_HBV _and more interconnected than the H-H network, indicated that HBV proteins have a strong tendency to interact with highly connected cellular proteins (Figure 1B, Additional file 1, Table S4). This also suggests that HBV preferentially targets host proteins already known to be engaged in protein-protein interactions.

### Analysis of the relationship between hepatocellular carcinoma and H_HBV_

In order to provide a global view of human proteins involved in HCC associated with the HBV - with the aim of clarifying the relationship between HBV proteins and hepatocellular carcinoma-associated proteins (H_HCC_) - we also made use of NLP methods to extract literature related to HCC from PubMed.

**Figure 2:**
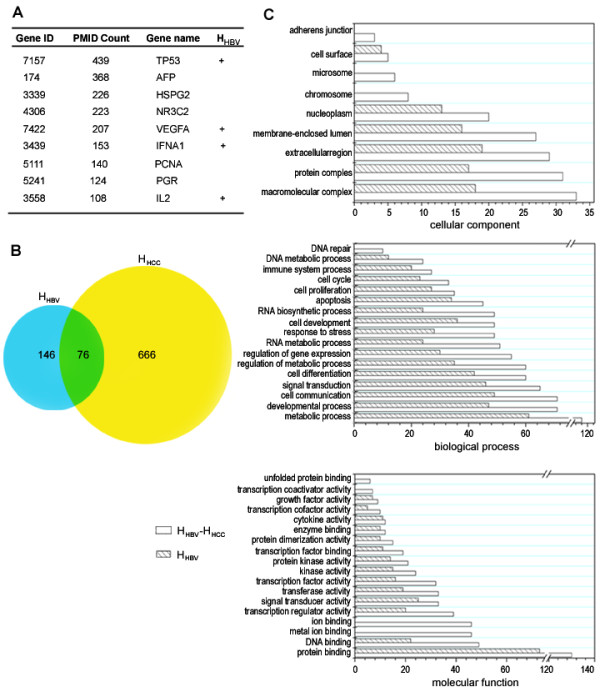
**Analysis of the relationship between H_HCC _and H_HBV_**. (A) Gene list of top nine H_HCC_. (B) Overlap between H_HCC _and H_HBV_. The blue area corresponds to H_HBV_; the yellow area, to H_HCC_: and the green area, to H_HBV_-H_HCC_. (C) Gene Ontology analysis of H_HBV _and H_HBV_-H_HCC_.

Using the keyword search [e.g., (liver cancer "[title] OR" hepatocellular carcinoma "[title] OR" Liver Neoplasm "[title] OR" Liver Neoplasms "[title] AND (" 1980/01/01 "[PDAT] :" 2009/01/01 "[PDAT]))], we retrieved 19,050 related articles. Based on a combination of text mining procedures and expert curation, a total of 666 H_HCC _(number of PMID greater than or equal to 2) were identified from 6,709 summary descriptions (Additional file 1, Table S5). Among these, nine of H_HCC _had more than 100 PMID references (Figure 2A).

Compared with H_HBV_, 76 proteins (H_HBV_-H_HCC_) among the H_HBV _(146) were also hepatocellular carcinoma-associated proteins (Figure 2B, Additional file 1, Table S6). Four H_HBV_-H_HCC_'s had more than 100 PMID references (Figure 2A).

### Gene ontology and KEGG pathway analysis

The 146 H_HBV _could be classified into 18 mutually dependent functional sets, resulting in 17 cellular processes in 12 cellular components according to the gene ontology analysis. Accordingly, the 76 H_HBV_-H_HCC _could be classified into 14 functional sets, resulting in 16 cellular processes in eight cellular components (Additional file 1, Table S7).

As shown in Figure 2C, most of the functional profiling showed transcriptional activity, DNA binding, kinase activity, signal transducer activity, cytokine activity and growth factor activity. These functions are very closely associated with developmental processes, cell differentiation, signal transduction, cell communication, apoptosis, regulation of gene expression, cell proliferation and cell development. These biological processes are thought to play important roles in the pathogenesis of HCC [2].

To better understand the biological functions of H_HBV_-H_HCC_, we determined the enrichment of specific pathways for all interactors. Of the 76 proteins (H_HBV_-H_HCC_), 63 (~83%) could be mapped to 9 pathways (P < 0.01) of 202 KEGG human pathway database (Additional file 1, Table S8). 6 pathways, namely apoptosis, cell cycle, p53 signaling pathway, toll-like receptor signaling pathway, MAPK signaling pathway and ErbB signaling pathway were significantly enriched (P < 0.0001).

### Functional analysis of the HBV-human interaction network

**Figure 3:**
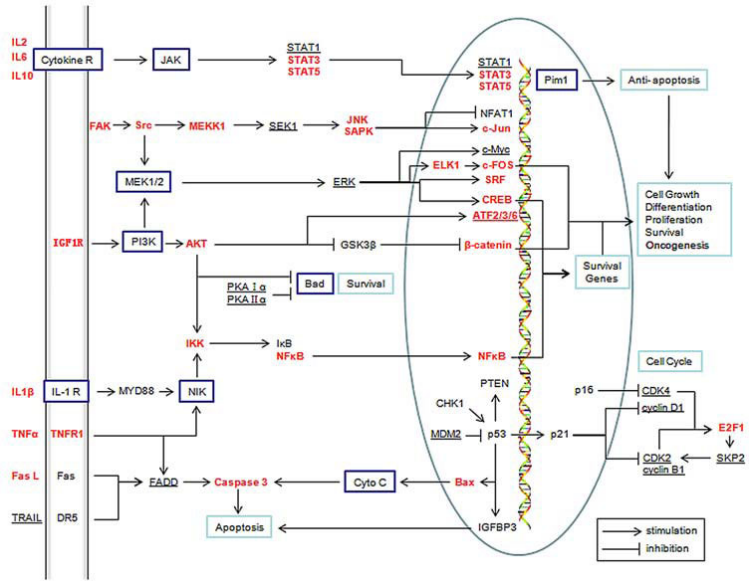
**Functional analysis of the HBV-human interaction network**. In black, H_HBV_-H_HCC _either down-regulated or inactivated; in red, H_HBV_-H_HCC _either up-regulated or overactivated; with underline, H_HBV_-H_HCC _interact (activate or inhibit unknown); in box, non-H_HBV_-H_HCC _molecules in pathways. See text for details.

Dysregulation of the balance of survival or apoptosis represents a protumorigenic principle in human hepatocarcinogenesis [20]. To provide a review of the current findings about how the balance is dysregulated by HBV in HCC, we integrated 57 H_HBV_-H_HCC _into one molecular interaction network. As shown in Figure 3, these H_HBV_-H_HCC _can constitute several signal pathways, such as JAK/STAT, MEK/ERK, PI3K/AKT, NFκB, MAPK, SAPK/JNK, and p53 signal pathways, and mediate many opposing cellular functions, including function in cell cycle and apoptosis regulation [21].

The expression of cytokines like IL2, IL6, TNF and receptors like insulin-like growth factor 1 receptor (IGF1R) are up-regulated, which can activate kinases like the Src tyrosine kinases and the downstream pathway such as MAPK, MEK/ERK. HBx activates the components of the JAK/STAT, MEK/ERK, PI3K/AKT, MAPK, SAPK/JNK signalling pathways, leading to activation of a variety of transcription factors such as STAT-3, ELK-1, NF-κB, CREB, β-catenin, c-Fos, c-Jun, c-Myc, etc. Meanwhile, some physiological proapoptotic molecules are down-regulated or inactivated, such as Fas, p53, DR5 or FADD. HBx can bind to the C-terminus of p53 sequesters in the cytoplasm and prevent it from entering the nucleus [2], failure to up-regulate genes, such as IGFBP3, p21_WAF1_, Bax or Fas, thereby inactivating several critical p53 dependent activities, including p53 mediated apoptosis. Moreover, the down-regulation of PTEN and the activation of PI3K/AKT-Bad pathway can inhibit TGFβ and FasL induced apoptosis and down-regulation of caspase 3 activity. However, HBx also promotes the apoptosis by regulating the expressions of Fas/FasL, Bax/Bcl-2, and c-Myc gene.

Cell cycle is regulated by activity of cyclins, cyclin dependent kinases (CDKs), and cyclin dependent kinase inhibitors (CDKIs), which generally function within several pathways [22]. HBx can repress the transcription of p21_WAF1 _and p16_INK4A_, leading to increase the rate and level of activation of the CDK2 and CDK4. HBx also inhibit the pRb tumor suppressor and increase E2F1 activity, and regulate the expression of MDM2, cyclin D1 and cyclin B1. Ultimately, HBx has been shown to stimulate cell cycle progression by accelerating transit through the G1/S and G2/M checkpoints [2].

In brief, regardless of the mechanism, the aberrant gene expression and deregulated of these pathways ultimately leads to generate a unique response, the acceleration of cell cycle progression and cell growth, increased differentiation and proliferation, repression of apoptosis, and contribute to cell survival and oncogenesis.

## Discussion

Developing an HBV-human interactome network by mapping the interactions of viral proteins with host proteins may give us a comprehensive view of viral infection at the protein level, and provide clues to understanding the development of end-stage complications such as cirrhosis and HCC. In this study, we used an NLP method to analyze the PubMed literature database for articles regarding HBV and human protein interactions. With an exhaustive analysis of the literature and databases, we identified 146 H_HBV _that are crucial for hepatitis B virus infections. These H_HBV _are involved in numerous functions associated with oncogenesis, and through screening and mapping the H_HCC_, we found that about half of the H_HBV _were also hepatocellular carcinoma-associated proteins such as IL6, STAT3[23], MMP9, TGFB1 [24] and TP53 [25]. This may explain why hepatitis B virus is the primary risk factor for the development of HCC. The Gene ontology analysis show that most of the functional profiling (such as transcriptional activity, DNA binding, kinase activity and signal transducer activity) and biological processes (such as cell differentiation, apoptosis, cell proliferation and cell development) are thought to play important roles in the pathogenesis of HCC.

KEGG functional annotation was used to analyze the biological functions of H_HBV_-H_HCC_. 83% of H_HBV_-H_HCC _could be mapped to 9 pathways (P < 0.01) (Additional file 1, Table S8), apoptosis, cell cycle, p53 signaling pathway, toll-like receptor signaling pathway, MAPK signaling pathway and ErbB signaling pathway were significantly enriched (P < 0.0001). Although this approach is biased because functions have not yet been attributed to all proteins, it remains a powerful way of incorporating conventional biology into systems-level data sets[26].

Toll-like receptors (TLRs) are known to play a key role in the innate immune system, particularly in the inflammatory response against invading pathogens [27]. In PBMCs of HBV-infected patients, TLR7 expression and TLR9 mRNA are down-regulated, but TLR9 shows increased protein expression [28], which may play important roles in cancer cells[29]. The ErbB receptor tyrosine kinases play important roles in both normal physiology and cancer. ErbB2 (HER-2/neu) has been identified as an important regulator of the metastatic potential of breast cancer, which is the principal cause of death [30]. The detailed relationship between HBV with ErbB receptor and toll-like receptors pathways has not been investigated. Further studies of the functional changes in these pathways in response to HBV infection will provide clear information about the oncogenesis of hepatocellular carcinoma.

We also identified focal adhesion (p < 0.001) might be as a novel pathway affected by HBV through the KEGG pathway analysis (Additional file 1, Table S8). When focal adhesion is deregulated, it can lead to perturbation of cell mobility, detachment from the ECM and tumor initiation and progression [31]. HBx can increase the migratory phenotype of hepatoma cells through the up-regulation of matrix metalloproteinases-1 (MMP1) and MMP9[32]. Moreover, HBx represses several cell adhesion molecules and cytoskeleton proteins, including E-cadherin, integrin, fibronectin, CD47, and CD44 [2]. Regulation of focal adhesion was also identified as a new function that is affected by HCV, primarily through the NS3 and NS5A proteins [26]. However, the impact of HBV protein on focal adhesion should be further assessed using a cellular adhesion assay.

Moreover, a large number of H_HBV_-H_HCC _could be significantly enriched in apoptosis, cell cycle, p53 and MAPK signaling pathway (P < 0.0001), which are very crucial in the oncogenesis of HCC [20]. Therefore, we integrated these H_HBV_-H_HCC _into one molecular interaction map, which delineate many different oncogenic pathways involved in hepatocarcinogenesis. These proteins are at the center of many different pathways (such as JAK/STAT, MEK/ERK, PI3K/AKT, NFκB, MAPK, SAPK/JNK, and p53 signal pathways) that regulate many important biological processes, including cell differentiation, apoptosis, cell proliferation, cell cycle, etc. HBx can modulate both pre-apoptotic and anti-apoptotic pathways, some physiological pro-apoptotic H_HBV_-H_HCC _molecules are down-regulated or inactivated, even more anti-apoptotic signals H_HBV_-H_HCC _molecule are up-regulated or over-activation [2]. Therefore, a significant number of the molecular events are altered, leading to the disruption of the balance between death and survival in the preneoplastic hepatocytes and the uncontrolled growth of tumour cell [20,21].

Accordingly, hepatocellular carcinoma show stronger requirements of these intracellular pathways to survive, therefore, therapeutic strategies to selectively inhibit anti-apoptotic signals in HCC cells might have the potential to provide effective tools to treat HCC in the future [4,20]. Interestingly, recently studies show that the multikinase inhibitor drug sorafenib can induce HCC apoptosis through inhibiting the RAF/MEK/ERK pathway [33]. Another receptor tyrosine kinase inhibitor drug, sunitinib is also a strong apoptosis inducer in different tumor cells, especially in the presence of inhibitors of the PI3K/Akt/mTOR pathway [34]. Similar situations might be found with other multikinase that are on the way towards approval for HCC therapy [34].

Therefore, the data of H_HBV _and the most specific annotations for each human protein can be used as a resource for researchers interested in prioritizing drug targets (Additional file 1, Table S1). For example, the damage-specific DNA binding protein 1 (DDB1) had 14 identified interactions with HBV X protein (Additional file 1, Table S1), which is a highly conserved protein implicated in DNA repair and cell cycle regulation [35]. HBx in association with DDB1 may stimulate HBV replication and induce genetic instability in hepatocytes, thereby contributing to HCC development, and making this HBV-host protein interaction as an attractive target for new therapeutic interventions [36].

In addition, it must be point out that not all of the papers that report HBV binding proteins from cell lines validate the binding of these host proteins to the corresponding HBV antigen by co-immunoprecipitation of extracts from clinical samples (infected liver and HCC tissue). At the same time, it raises a number of questions need further studies such as whether all the identified interactions really occur and have functional consequences. To identify new molecules involved in hepatocarcinogenesis, we can establish of high-throughput yeast two-hybrid (Y2H) screens and co-affinity purification methods for large scale analysis of protein-protein interaction[26], and integrate of chip-based chromatin-immunoprecipitation (ChIP-chip) with expression-microarray profiling for the identification of candidate genes directly regulated by HBV[37].

Finally, a number of H_HBV_-H_HCC _and cellular processes have been studied, but many of the molecular events involved in the pathophysiology of HCC are still unclear. One single identified H_HBV_-H_HCC _may be involved in some new multiple, independently regulated HCC-specific pathways. Hence, the HBV-human protein interaction network might be to regard as the basis of a detailed map for tracking new cellular interactions, and guiding future investigations of the molecular mechanism of oncogenesis of HBV-related HCC, even other diseases such as steatosis and fibrosis, leading to identify a series of new genes involved in these diseases.

In mammals, lethal and disease-related proteins were found enriched among some proteins that are central to multiple pathways [38,39], and preferential attachment to these proteins may be a general hallmark of viral proteins, as has recently been suggested in an analysis of the literature [40]. An important breakthrough of the further experimental study is the identification of novel signaling components and pathways that can be targeted to develop new therapeutics.

## Conclusions

Among the infectious diseases affecting humans, HBV is one of the most common diseases in the world, particularly in developing countries. More than 350 million people worldwide are known to be chronic carriers of HBV, and each year 15 million people die of hepatitis. HCC is one of the most common fatal cancers worldwide, and the incidence of HCC in many countries is increasing in parallel to an increase in chronic HBV infection. Because the role of HBV infection and the pathogenic mechanisms of the cancer-causing variant are not entirely clear, there is still a lack of effective treatment of HCC. For an in-depth review and understanding of these interactions, to enhance insight into HBV replication and pathogenesis on a cellular level, we catalogued all published interactions between HBV and human proteins, particularly human proteins associated with HCC. We have provided a general overview of the landscape of human proteins that interact with HBV.

## Competing interests

The authors declare that they have no competing interests.

## Authors' contributions

ZJW and YZ made substantial contributions to conception and design, acquisition of data, analysis and interpretation of data; DRH involved in drafting the manuscript; ZQW conceived of the study, and participated in its design and drafted the manuscript. All authors read and approved the final manuscript.

## Supplementary Material

Additional file 1 **Additional Tables**. Table S1. Total interactions between HBV and human proteins catalogued from related literature. The meaning of each is as follows: Pubmed_ID: PubMed article ID. HBV_gene_mention: HBV gene name appeared in the sentence. HBV_gene: the HBV gene after standardization. verb_mention: the meaning of the verb or verb noun such as heavier appeared in the sentence. verb: the verb after standardization. human_gene_mention: human gene names appeared in the sentence. human_official_gene_symbol: the human gene after standardization. human_gene_entrez_ID: standardization of the ID of the human gene. human_official_gene_description: standardization of the description of the human gene. sentence: the key sentence. PubMed_link: PubMed abstract link. Additional file 1, Table S2. Listing and Distribution of Keywords Associated with the HBV Human Protein Interaction Database. Statistical analysis of interaction verb and calculation of the proportion of each verb. Additional file 1, Table S3. Listing of human proteins interacting with more than one viral protein. Additional file 1, Table S4. Listing of H_HBV_-H_HBV _protein- protein interactions. Interacting human proteins are referenced with their cognate NCBI gene name (columns 1 and 2). These physical and direct binary protein-protein interactions were retrieved from the BIND, BioGRID, DIP, GeneRIF, HPRD, IntAct, MINT, and Reactome databases. Interaction type (6 = KEGG database,7 = text mining,8 = homology). Additional file 1, Table S5. Hepatocellular carcinoma-associated proteins (H_HCC_) catalogued from related literature. Additional file 1, Table S6. Listing of H_HBV_- H_HCC_. H_HBV_: HBV-interacting proteins. H_HCC_: liver cancer-related genes. H_HBV_- H_HCC_: overlap. Additional file 1, Table S7A. Distribution of cellular component Gene Ontology terms associated with HBV-human protein interactions. Additional file 1, Table S7B. Distribution of biological process Gene Ontology terms associated with HBV-human protein interactions Additional file 1, Table S7C. Distribution of molecular function Gene Ontology terms associated with HBV-human protein interactions Additional file 1, Table S8. Functional analysis of the H_HBV _distribution and enrichment in cellular pathways using KEGG annotations. Click here for file
